# Supplementary material for: Examining and investigating the impact of demographic characteristics and chronic diseases on mortality of COVID-19: Retrospective study
Source: PLoS One. 2021 Sep 10;16(9):e0257131. doi: 10.1371/journal.pone.0257131 (PMC8432755; doi:10.1371/journal.pone.0257131)
Supplement: Attachment — Submitted filename: Response to Revierers.docx [file pone.0257131.s002.docx]

5 April 2021

Dear Editor,

# **Re: Revised Manuscript ID PONE-D-21-01309**

Please find attached our revised manuscript: “Examining and Investigating the Impact of Demographic Characteristics and Chronic Disease with Mortality of COVID-19: Retrospective study in PLOS ONE. We would like to thank the reviewer for their thoughtful consideration of our manuscript.

| **Comments** | **Authors’ Response** |
| --- | --- |
| **Comments to the authors** | |
| 1. Is the manuscript technically sound, and do the data support the conclusions?  The manuscript must describe a technically sound piece of scientific research with data that supports the conclusions. Experiments must have been conducted rigorously, with appropriate controls, replication, and sample sizes. The conclusions must be drawn appropriately based on the data presented.  Reviewer #1: Partly  Reviewer #2: Partly | The manuscript has now been re-worked, data has been reanalysed as per the reviewers comments and suggestions. |
| 2. Has the statistical analysis been performed appropriately and rigorously?  Reviewer #1: No  Reviewer #2: Yes | Data has been completely re-analysed |
| 3. Have the authors made all data underlying the findings in their manuscript fully available?  The PLOS Data policy requires authors to make all data underlying the findings described in their manuscript fully available without restriction, with rare exception (please refer to the Data Availability Statement in the manuscript PDF file). The data should be provided as part of the manuscript or its supporting information, or deposited to a public repository. For example, in addition to summary statistics, the data points behind means, medians and variance measures should be available. If there are restrictions on publicly sharing data—e.g. participant privacy or use of data from a third party—those must be specified.  Reviewer #1: No  Reviewer #2: No | Data is fully available |
| 4. Is the manuscript presented in an intelligible fashion and written in Standard English?  PLOS ONE does not copyedit accepted manuscripts, so the language in submitted articles must be clear, correct, and unambiguous. Any typographical or grammatical errors should be corrected at revision, so please note any specific errors here.  Reviewer #1: Yes  Reviewer #2: Yes | Thank you |
| **Reviewer 1** | |
| Abstract: The background does not included the mortality situation of COVID-19 in Saudi Arabia. Additionally, it is interesting to mention the place and time period of data collection. | Edited |
| 1) In the introduction, I would suggest some references mentioning the situation of covid-19 mortality in Saudi Arabia. Furthermore, citing the relationship between the disease, other comorbidities, and the socio-demographic characteristics. | We have rewritten the introduction utilising new references mentioning the situation of COVID-19 risk factors associated with morality in Saudi Arabia. |
| 2) In Methods, correct the sentence: "Data were recruited from April 2020 to June  202, data collection included reviewing patients’ electronic medical records, nursing notes, laboratory characteristics, management details and clinical outcomes.” | Corrected |
| 3) The authors report that they collected data about “patients’ electronic medical records, nursing notes, laboratory characteristics, management details and clinical outcomes”. Why were not all these data used in the statistical analysis? | The aim of the study of the study was to investigate whether demographic characteristics (age and gender) and non-communicable diseases (hypertension (HTN) and diabetes mellitus (DM)) have significant association with mortality in COVID-19 patients. Therefore, the data which support the study aim were included. |
| 4) In the data analysis, I suggest describing the variables compared to the mortality group (yes or no). These comparisons should evaluate using a statistical test. Moreover, indicate which program was performed the statistical analysis. | Data has been re-analysed to get the suggested comparisons results based on reviewer comment (TQ) |
| 5) In the Results section, I suggest highlight the most important findings and joining tables 1 and 2. In addition, I suggest placing all comparisons between mortality groups in this unified table. | Tables 1 and 2 has been joined to get the best findings based on reviewer comments (TQ) |
| 6) In Table 3, I would show the logistic regression (crude and adjusted) for the variables in the joined table. I suggest presenting only odds ratio (OR), confidence interval (CI 95%), and p-value. | Table 3 has been amended based on reviewer comment (TQ) |
| 7) In the Table 5, I would show only odds ratio (OR), confidence interval (CI 95%), and p-value. | Table 5 has been amended based on reviewer comment (TQ) |
| 8) The Discussion section will need review, including other references. I would suggest better elaborating on the limitations of the study. For instance, I would include the sample size, and other comorbidities. | We enhanced the discussion utilising new references, also we redeveloped the limitation section as per the reviewer comment. |
| **Reviewer 2** | |
| Title - Authors should consider 'impact on' instead of 'impact with' | Amended as per the reviewer comment |
| Methods - Line 2: Authors should take off 'have been employed' , Line 3: replace 'connection' with 'association' | Amended as per the reviewer comment |
| Design - line 3: Authors should check spelling of 'reviewing'. Line 7- Authors should clarify collection of personal data | Amended as per the reviewer comment |
| Data Analysis - Analysis is incomplete to draw conclusion on the association of demographic characteristics with morbidity among COVID-19 patients. Other demographic characteristics though not of interest to authors could also have been included in the study and controlled for, to better illustrate the impact of the demographic characteristics of interest to the authors. eg. Educational status, marital status, occupation status etc. Thus, the analysis raises important concerns and questions about what informed the inclusion of only some variables in the analysis? | Data has now been completely re-analysed as per the reviewer comments and suggestions to draw better conclusion on the association of demographic characteristics with morbidity among COVId-19 patients. |
| Table 1 - Why baseline? Since there is no end line data presented? Also, authors should consider using ;age(years) and state only numbers in the columns and avoid repeating 'years' in the column in the table in addition to the figures | Table 1 has been amended based on reviewer comment (TQ) |
| Table 3 -Authors should state the exact p-values. Authors should clarify their level of significance - is it <0.05 or >0.0005? Authors should state p-values to 3 decimal places. | Table 3 has been amended as per the reviewer comment (TQ) |
| Table 4- Some p-values are stated in exact figures while others are not. Mixture does not sound statistically acceptable. | Data has been re-analysed to get the suggested results based on reviewer comment (TQ) |
| Table 5 -Analysis is not clear, what is the objective of analyzing HPT VS. DM? Do the authors mean HPT & DM instead? | Table 5 has been amended as per the reviewer comment (TQ) |

**Thank you once again for the feedback received, and I hope that this information fulfils all of your requirements. Should you have any queries or require further information please do not hesitate to contact me for clarification**.
