# Supplementary material for: Examining and investigating the impact of demographic characteristics and chronic diseases on mortality of COVID-19: Retrospective study
Source: PLoS One. 2021 Sep 10;16(9):e0257131. doi: 10.1371/journal.pone.0257131 (PMC8432755; doi:10.1371/journal.pone.0257131)
Supplement: Attachment — Submitted filename: Response to Revierers.docx [file pone.0257131.s003.docx]

1 May 2021

Dear Editor,

# **Re: Revised Manuscript ID PONE-D-21-01309**

Please find attached our revised manuscript: “Examining and Investigating the Impact of Demographic Characteristics and Chronic Disease with Mortality of COVID-19: Retrospective study in PLOS ONE. We would like to thank the reviewer for their thoughtful consideration of our manuscript.

| **Comments** | **Authors’ Response** |
| --- | --- |
| **Comments to the authors** | |
| **Reviewer 1** | |
| Reviewer #1: 1) In the Introduction section, correct the sentence: “Globally, as of 6 April 2021, there have been 131,020,967confirmed cases of COVID-19, including 2,850,521 deaths, reported to the World Health Organization.” | Corrected |
| 2) In table 1 (Results section), I would show totals column and round percent values. The comparisons between ‘survivors’ and ‘non-survivors’ should be evaluate using a statistical test. | This table created to show the demographic characteristics that have been grouped under survivors (recovered) and non-survivors (died) as demonstrated |
| 3) Indicate which program was performed the statistical analysis. | Indicated |
| 4) In tables 2, 3 and 4 change “sig.” by “p-value”. Additionally, the column order is Variable name, Odds Ratio (OR), CI 95%, and p-value. If the exact p-value is less than 0.001, it is conventional to state merely ‘p < 0.001’. Constant value is not necessary in the logistic regression. Include “adjusted” term in tables 2 and 3. | Corrected |
| 5) I do not understand the “@” term in the results comments. | Corrected  Our significance level at the rate (@) confident that 95 out of 100 times the estimate will fall between the upper and lower values specified by the confidence interval.  For example CI(1.036, 1.103) Lower value is 1.036  Higher value is 1.103 |
| 6) The findings of the ‘Age’ and ‘Gender’ variables in the text are different of the Table 2. | Corrected |
| 7) Change OD by OR in the sentence “The results of logistic regression showed that the history of HTN [OD=1.822,P<0.05] @ CI [1.64,5.21] and DM [OD=4.011,P>0.05] @ CI [1.523,10.561] have significant association with mortality” | Corrected |
| 8) In the Discussion section, I didn’t find references in the results about the sentence “The binary logistic regression analysis used in our study quantitatively suggests that the COVID-19 patients who have age >50 years, who are males, or who have history of HTN and/or DM may have up to 9-fold increased chances of mortality than those patients who are young, who are females and are non-diabetic and normotensive”. The logistic regression was carried out with a continuous age variable, but the discussion focuses on the findings in older patients | This has been reported in the results section please refer to the following sentence “as demonstrated in Table 2. The findings demonstrated that age [OR=1.08, *P* <0.05] at CI [1.036 - 1.103] and gender [OR=5.939, *P* < 0.05] at CI [2.084 - 16.921] have significant association with mortality. The odds of non-survivors are 1.069 times among older patients as compared to younger. According to gender, the odds of non-survivors among male patients are 2.084 times as compared to female patients. In other words, older patients more likely to die as compared to younger patients. Besides that, males patients more likely to die due to COVID-19 virus as compared to female patients” |
| 9) Explain how the selection bias that occurred in this study | Explained, please refer to the manuscript |

**Thank you once again for the feedback received, and I hope that this information fulfils all of your requirements. Should you have any queries or require further information please do not hesitate to contact me for clarification.**
