# Supplementary material for: Examining and investigating the impact of demographic characteristics and chronic diseases on mortality of COVID-19: Retrospective study
Source: PLoS One. 2021 Sep 10;16(9):e0257131. doi: 10.1371/journal.pone.0257131 (PMC8432755; doi:10.1371/journal.pone.0257131)
Supplement: Attachment — Submitted filename: Rebuttal letter.docx [file pone.0257131.s004.docx]

24 June 2021

Dear Editor,

# Re: Revised Manuscript ID PONE-D-21-01309

Please find attached our revised manuscript: “Examining and Investigating the Impact of Demographic Characteristics and Chronic Disease with Mortality of COVID-19: Retrospective study in PLOS ONE. We would like to thank the reviewer for their thoughtful consideration of our manuscript.

| **Comments** | **Authors’ Response** |
| --- | --- |
| **Comments to the authors** | |
| 1. If the authors have adequately addressed your comments raised in a previous round of review and you feel that this manuscript is now acceptable for publication, you may indicate that here to bypass the “Comments to the Author” section, enter your conflict of interest statement in the “Confidential to Editor” section, and submit your "Accept" recommendation.  Reviewer #1: All comments have been addressed | Thank you |
| 2. Is the manuscript technically sound, and do the data support the conclusions?  The manuscript must describe a technically sound piece of scientific research with data that supports the conclusions. Experiments must have been conducted rigorously, with appropriate controls, replication, and sample sizes. The conclusions must be drawn appropriately based on the data presented.  Reviewer #1: Partly | Thank you |
| 3. Has the statistical analysis been performed appropriately and rigorously?  Reviewer #1: Yes | Thank you |
| 4. Have the authors made all data underlying the findings in their manuscript fully available? The [PLOS Data policy](https://eur01.safelinks.protection.outlook.com/?url=http%3A%2F%2Fwww.plosone.org%2Fstatic%2Fpolicies.action%23sharing&data=04%7C01%7Camutairi%40taibahu.edu.sa%7C9c432071ceac424acc8408d93662fc67%7C13a8d02d59f3416a8231b3080e639cad%7C0%7C0%7C637600621036439006%7CUnknown%7CTWFpbGZsb3d8eyJWIjoiMC4wLjAwMDAiLCJQIjoiV2luMzIiLCJBTiI6Ik1haWwiLCJXVCI6Mn0%3D%7C1000&sdata=jcGE1pZpIzCTagMITni2%2FKfligUwv0O1pyoVbv8fVkk%3D&reserved=0) requires authors to make all data underlying the findings described in their manuscript fully available without restriction, with rare exception (please refer to the Data Availability Statement in the manuscript PDF file). The data should be provided as part of the manuscript or its supporting information, or deposited to a public repository. For example, in addition to summary statistics, the data points behind means, medians and variance measures should be available. If there are restrictions on publicly sharing data—e.g. participant privacy or use of data from a third party—those must be specified.  Reviewer #1: Yes | Thank you |
| 5. Is the manuscript presented in an intelligible fashion and written in standard English?  PLOS ONE does not copyedit accepted manuscripts, so the language in submitted articles must be clear, correct, and unambiguous. Any typographical or grammatical errors should be corrected at revision, so please note any specific errors here.  Reviewer #1: Yes | Thank you |
| **Reviewer 1** | |
| Reviewer #1: 1) In the Introduction section, correct the sentence: “Another unique prognostic factor in Saudi Arabia is the migrant labor workers who are mostly men coming from less developed countries and known of increasing of increasing prevalence of cardiometabolic diseases.” | Corrected |
| 2) Include bibliography reference about SPSS program | Included, please refer to the manuscript |
| 3) In tables 2, 3 and 4 change “Exp(B)” by “Odds Ratio (OR)”. Additionally, the column order is Variable name, Odds Ratio (OR), 95% CI, and p-value. If the exact p-value is less than 0.001, it is conventional to state merely ‘p < 0.001’. Constant value is not necessary in the logistic regression. Include “adjusted” term in tables 2 and 3. | Table 2, 3 and 4 were fixed as per the reviewer comment. |
| 4) I do not know the ‘@’ term in the confidence interval. I think that the correct term is “OR=2.77 (95% CI 1.60 - 4.78)”, for example. Furthermore, use this sentence for comment the results. So, you do not need to use p-value together with 95% CI. | Edited as per the reviewer comment, please refer to the results section |

**Thank you once again for the feedback received, and I hope that this information fulfils all of your requirements. Should you have any queries or require further information please do not hesitate to contact me for clarification**.
