# Supplementary material for: Examining and investigating the impact of demographic characteristics and chronic diseases on mortality of COVID-19: Retrospective study
Source: PLoS One. 2021 Sep 10;16(9):e0257131. doi: 10.1371/journal.pone.0257131 (PMC8432755; doi:10.1371/journal.pone.0257131)
Supplement: Attachment — Submitted filename: Rebuttal letter.docx [file pone.0257131.s005.docx]

29 July 2021

Dear Editor,

# Re: Revised Manuscript ID PONE-D-21-01309R3

Please find attached our revised manuscript: “Examining and Investigating the Impact of Demographic Characteristics and Chronic Disease with Mortality of COVID-19: Retrospective study in PLOS ONE. We would like to thank the reviewer for their thoughtful consideration of our manuscript.

| **Comments** | **Authors’ Response** |
| --- | --- |
| **Comments to the authors** | |
| **Reviewer 1** | |
| Reviewer #1: 1) In the Results section, correct all “OR” and “95%CI” value sentences with three decimal places. | Edited as per the reviewer comment. |
| 2) In tables 2 and 4 replace the p-values “0.0005” with “< 0.001”. | Edited as per the reviewer comment. |
| 3) In the Discussion section, I don't think it's interesting to put the term "etc" in scientific articles. Furthermore, in the last paragraph of the discussion section, include the bibliographic references | Edited improved as per the reviewer comment. |

Thank you once again for the feedback received, and I hope that this information fulfils all of your requirements. Should you have any queries or require further information please do not hesitate to contact me for clarification.
